# Allelic Gene Structure Variations in *Anopheles gambiae* Mosquitoes

**DOI:** 10.1371/journal.pone.0010699

**Published:** 2010-05-19

**Authors:** Jun Li, Jose M. C. Ribeiro, Guiyun Yan

**Affiliations:** 1 Department of Microbiology, University of Minnesota, St. Paul, Minnesota, United States of America; 2 Vector Biology Section, Laboratory of Malaria and Vector Research, National Institute of Allergy and Infectious Diseases, National Institutes of Health, Bethesda, Maryland, United States of America; 3 Program in Public Health, University of California Irvine, Irvine, California, United States of America; Centre de Regulació Genòmica, Spain

## Abstract

**Background:**

Allelic gene structure variations and alternative splicing are responsible for transcript structure variations. More than 75% of human genes have structural isoforms of transcripts, but to date few studies have been conducted to verify the alternative splicing systematically.

**Methodology/Principal Findings:**

The present study used expressed sequence tags (ESTs) and EST tagged SNP patterns to examine the transcript structure variations resulting from allelic gene structure variations in the major human malaria vector, *Anopheles gambiae*. About 80% of 236,004 available *A. gambiae* ESTs were successfully aligned to *A. gambiae* reference genomes. More than 2,340 transcript structure variation events were detected. Because the current *A. gambiae* annotation is incomplete, we re-annotated the *A. gambiae* genome with an *A. gambiae*-specific gene model so that the effect of variations on gene coding could be better evaluated. A total of 15,962 genes were predicted. Among them, 3,873 were novel genes and 12,089 were previously identified genes. The gene completion rate improved from 60% to 84%. Based on EST support, 82.5% of gene structures were predicted correctly. In light of the new annotation, we found that ∼78% of transcript structure variations were located within the coding sequence (CDS) regions, and >65% of variations in the CDS regions have the same open-reading-frame. The association between transcript structure isoforms and SNPs indicated that more than 28% of transcript structure variation events were contributed by different gene alleles in *A. gambiae*.

**Conclusions/Significance:**

We successfully expanded the *A. gambiae* genome annotation. We predicted and analyzed transcript structure variations in *A. gambiae* and found that allelic gene structure variation plays a major role in transcript diversity in this important human malaria vector.

## Introduction

Transcript structure variations (TSVs) are common in eukaryotic organisms [1], [2]. TSVs increase protein diversity and affect protein regulation. TSVs result from allelic gene structure variations and post-transcriptional alternative splicing (AS). It has been estimated by traditional molecular cloning methods that about 5% of genes in eukaryotic organisms experienced AS [3]. However, more than 42% of genes in humans were reported to experience AS, based on EST data [4]. Applying pooled mRNA on an exon-junction array indicated that up to 75% of human genes have transcript isoforms [5]. A recent survey on TSVs using data from the databases of dbSNP and cDNA that were generated from the same human individual [6] indicated that about 6% of TSVs are allele-specific [7].

Systematic study of TSVs has not been reported in *Anopheles gambiae*, the most important vector of human malaria, which kills millions of people annually. Several EST sequencing projects have been conducted in *A. gambiae* [8], [9]. These ESTs were used to predict genes [10]. Only 1,149 TSVs in 473 genes were presented in the AgamP3.4 database, which is apparently an under-prediction compared to more than 3,000 TSVs in *Drosophila* [11].

Allelic variations affect organisms in many ways. For example, several genetic loci responsible for malaria parasite resistance have been discovered in *A. gambiae* [12], [13], [14]. Allelic genetic variations are believed to be responsible for resistance to malaria parasite infection. However, finding the genetic variations that cause the parasite resistance is difficult because there are thousands of potential candidate genes at the loci. Genes with allelic structure variations are apparently the best candidates for direct and in-direct association studies for parasite resistance.

Meanwhile, AS, another important physiological event, can be predicted when a large set of true AS genes and sequences is available [15]. But the lack of data sets for training and verifying AS prediction algorithms has made prediction not very successful so far [16], [17]. Therefore, it is both theoretically and practically important that we are able to separate the AS events from allelic gene structure variations in TSVs. SNPs are very common in eukaryotic organisms. For instance, there is one SNP in every 100 bp on average in *A. gambiae* [10]; therefore, it is expected that different gene alleles have different SNP patterns. Because SNPs and mutations that cause allelic-specific transcript structure variations are in the same genes and thus tightly linked, we can develop an integrated computational tool that predicts the allelic gene structure variations based on ESTs alone.

TSV changes the protein amino acid sequence when variable regions occur within coding sequence (CDS) regions. Variations in CDS will change the protein sequences and further alter protein structures and functions. The variable regions at the 5′-untranslated regions (UTR) may change the protein expression level while the variable regions at the 3′-UTR may affect the mRNA stability and mRNA turnover rate. Therefore, accurate genome annotation is essential to correctly determine the effect of allelic gene structure variations on proteins.

The *A. gambiae* genome annotation is incomplete, since gene predictions were generated as a consensus of automated pipeline results from Celera Otto [18] and ENSEMBL tools [10]. Both pipelines relied on the Genewise comparative algorithm and other comparative data sources for gene and protein prediction. Comparative algorithms are inherently conservative because of their reliance on protein homology with other organisms, and they yield predictions with higher specificity but lower sensitivity [19]. Comparative algorithms will thus particularly miss genes that display rapid evolutionary rates, including mosquito-specific genes that could control responses to mosquito-specific pathogens like malaria, or genes involved in human host-seeking or blood feeding. In addition to under-prediction, comparative algorithms are known to have trouble predicting start/stop codons in flanking regions [19]. This will cause problems in identifying the variation location. A previously reported method of synthesizing the *ab intio* gene prediction algorithm and the comparative algorithm resolved the problem of incompletion [20]. However, over-prediction was observed because the human gene model, not the *A. gambiae* species-specific gene model, was used. In this paper we achieve a higher completion rate without over-prediction by using *A. gambiae* species-specific gene model in the combinational method.

## Results

### Transcript structure variations

About 234,004 unique ESTs were collected from publicly available databases. More than 80% of them were successfully aligned to the *A. gambiae* genome with a high percentage of coverage (>80%) and identity (>95%). Compared to the results in AnoEST [21], more ESTs were used while fewer EST clusters were formed. Using the alignment tools GMAP and GeneSeqer, 18,015 and 19,381 transcript clusters were obtained respectively based on alignments. In these transcript clusters, 9,426 and 6,903 total TSV events were detected in 3,210 and 2,341 clusters, respectively. Intersection of the two results from two different tools removed the false positives due to inaccurate sequence alignments. Consequently, 2,340 common variation events in 1,490 genes were identified. These common variations represent the lower boundary of variation events in *A. gambiae* because the intersection of two sets also removed many true positive TSVs. As a good representative set in *A. gambiae*, this data set was analyzed further.

**Figure 1:**
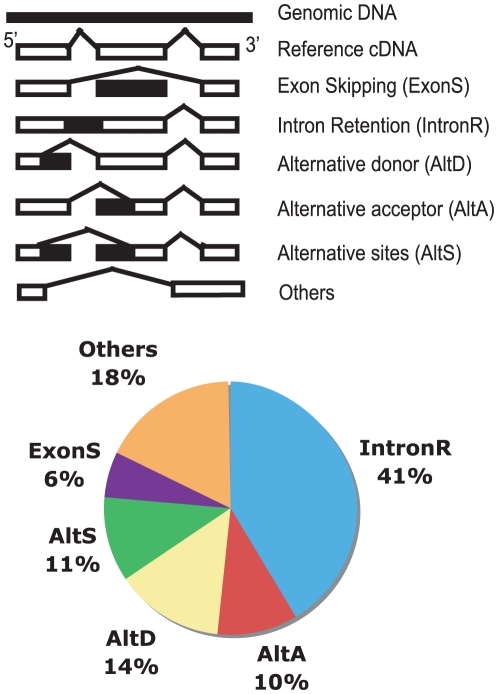
Transcript structure variations of *Anopheles gambiae* mosquitoes. **Upper panel** is a diagram illustrating the variation types. White boxes are exons in mRNA, and black boxes are parts of exons in some mRNA and parts of introns in others. The sites connected by lines are splicing sites. The “Others” type includes alternative transcript initialization, major gene structure changes or types that cannot be summarized by the other five categories. **Lower panel** shows the frequency distribution of transcript structure variation types in *A. gambiae*.

As shown in the lower panel of Fig. 1, 41% of the variation events were intron retention (IntronR), 10% were alternative acceptors (AltA), 14% were alternative donors (AltD), 11% were alternative donors and acceptors (AltS), and only ∼6% of variation events were exon skippings (ExonS) in *A. gambiae*. In addition, 18% of TSVs could not be classified in the above five categories. The “Others” category included various cases such as alternative transcript initialization and complicated structure changes. Because the variations in “Others” had many different subtypes and their structures were difficult to classify, we did not include this type for further analysis in this paper.

### Genome annotation

The impact of gene structure variations depends on their locations in either CDS or un-translated regions. The *A. gambiae* genome annotation (AgamP3.4) is far from complete due to the limitation of the prediction method. We predicted genes using combinational algorithms as reported previously [20]. Improving on the previous prediction, we used GlimmerHMM [22] trained with the *A. gambiae* specific gene model as the *ab initio* gene prediction tool. A total of 15,962 genes were found, more than AgamP3.4 annotation (∼12,457). This set was named ReAnoGene09. Among our predicted genes, 89.6% of genes on placed chromosomes (n = 14,662) were supported by ESTs, and only 44% of genes on unplaced contigs (termed as UNKN chromosome, n = 1,300) were supported by ESTs. Manual verification indicated that about 82.5%±12.5% of gene structures were annotated correctly according to the EST support.

**Figure 2:**
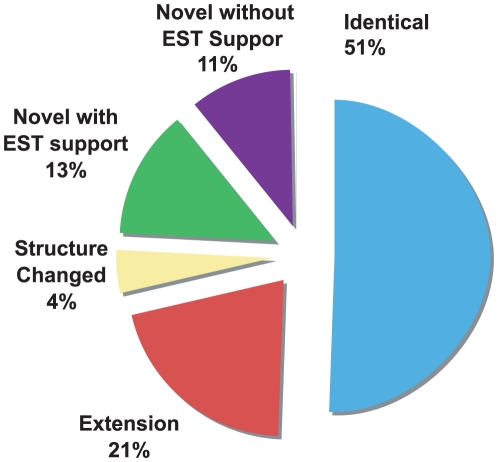
Comparison between the newly annotated CDS and AgamP3.4 CDS. We expanded the *Anopheles gambiae* annotation by increasing the completion rate through extension of genes and discovered more than 2,000 EST-supported novel genes without over-prediction.

When the CDS structure of ReAnoGene09 is compared to the AgamP3.4 annotation, 8,062 genes were identical, 3,323 genes were extension forms of the AgamP3.4 gene, and 704 genes had internal structure changes. In addition, 3,873 genes were novel genes, 55% of which were supported by ESTs (Fig. 2). Our annotation was based on AgamP3.4 annotation. The recent AgamP3.5 annotation release (Sept 2009 release) was significantly different from AgamP3.4 (>40% CDS changed). Comparing the CDS structure of ReAnoGene09 with AgamP3.5, we found that 7,326 genes were identical, and 3,052 ReAnoGene09 genes were novel. This indicates that ReAnoGene09 expanded the *A. gambiae* genome annotation. The completion rates of AgamP3.4 and AgamP3.5 (Sept 2009 release) annotation were about 60% and 79% respectively. We increased the completion rate by finding the correct initial and terminal exons. The completion rate of CDS in the newly annotated genes was more than 84% (n = 13,426). The median CDS length is 927 bp, while the average length of 5′-UTR and 3′-UTR is about 234 bp and 451 bp respectively. Considering the number of transcript structure variations detected in this paper, it is worthwhile to note that the total number of CDS and protein forms could be double the gene number.

### The impact of TSVs on coding

**Figure 3:**
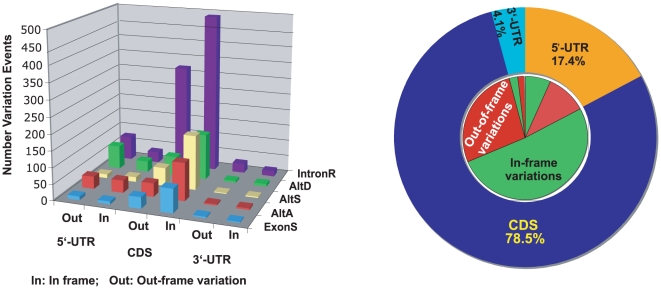
Transcript structure variation (TSV) locations and length difference in *Anopheles gambiae*. **Left panel** shows the detail of variation locations and length differences in different TSV types. For all TSV types, the open reading frames of variations within CDS regions are conserved. **Right panel** shows the summary of variation locations and length differences. The green and red colors in the inner circle represent in-frame and out-of-frame variations respectively. More than 65% of variations at CDS regions are in-frame, while variations at un-translated regions (UTR) tend to be out-of-frame. Therefore, most transcript structure variations just insert or delete some amino acids without changing the protein structures. The data also show that more variations are at 5′-UTR than at 3′-UTR, which suggests the higher efficiency on gene regulation at 5′-UTR than 3′-UTR.

Under the guide of the new genome annotation obtained above, 78.1% of TSVs were found to be in CDS regions (Fig 3). The length changes at CDS regions affect their downstream open reading frame (ORF). The chance of introducing an early terminator at a CDS region by out-of-frame changes (the length difference between isoforms is not divisible by 3) was much higher than that by in-frame (the length difference between isoforms is divisible by 3) changes. Early terminators might cause nonsense-mediated mRNA decay (NMD) [23]. Therefore, we calculated the length differences between variable isoforms. Results indicated that the majority of variations at CDS regions for all variation types were in-frame (Fig. 3 left panel). This observation is consistent with reports in other organisms [11]. Manual verification of variation at CDS regions indicated that many out-of-frame variations at CDS regions were near the end of the CDS (∼48%), or the original open reading frames were restored by nearby mutations. Therefore, most transcript structure variations just insert or delete some amino acids or functional motifs without changing the whole protein sequence and structure. Since the variations at the UTR don't change the protein coding, the frequency of in-frame variations at the UTR is close to the random rate.

In addition, we also observed 17.7% of variations at 5′-UTR and 4.2% at 3′-UTR (Fig. 3). Since the median length of 5′-UTR is shorter than that of 3′-UTR (see materials and methods), the higher variation frequency at 5′-UTR is proposed to be related to higher efficiency on gene regulation at 5′-UTR than 3′-UTR.

### *In silico* detection of TSV source

Transcript structure variation comes from allelic variations and AS. SNPs and structural variations on the ESTs are on the same genes; therefore, their genotypes are tightly linked. Since AS isoforms are expected from the same allele, and allelic gene structure variations are from different alleles, we used the EST associated SNPs to distinguish the allelic gene structure variations from AS.

There were 340,588 SNPs present in ESTs. Considering that the sequencing error rate in ESTs was estimated to be about 0.0044 per base pair [24], there would be about one false positive SNP in a 227-bp EST. To reduce the false positive rate, we used the SNPs that were found in more than one EST. In total, 113,367 SNPs were present in more than one EST (the set of multi-hit SNPs is called mSNP in this paper). There was less than 1 false positive per 50 kb in mSNP.

**Figure 4:**
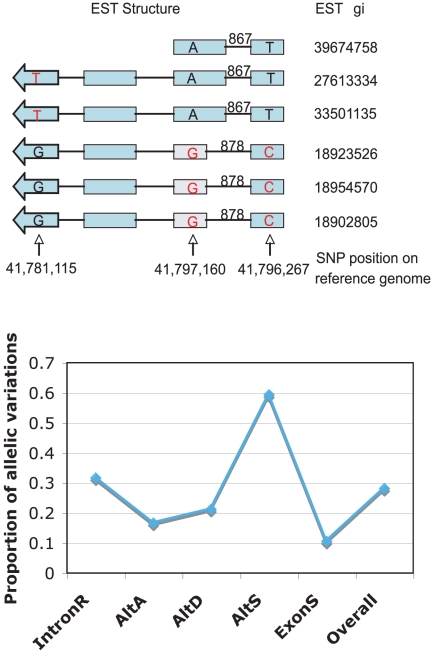
Distinguishing allelic gene structure variations from alternative splicing. **Upper panel** shows a diagram using SNP on EST as tags to distinguish allelic variations from alternative splicing with one AltA case. The light boxes are exons, the lines linking these boxes represent introns, and the numbers above the lines are intron length. The letters within boxes indicate the SNPs at a specific position labeled in different ESTs (gi numbers are shown on the right). In the example here, the SNP pattern T-A-T is associated with one transcript isoform, while SNP pattern G-G-C is associated with another transcript isoform. SNPs existing in multiple ESTs were used to distinguish the allelic variations from alternative splicing (see methods), and ESTs that didn't have mSNP were removed from the estimations. **Lower panel** shows the frequency distribution of allelic gene structure variations in TSVs based on this method.

Not all ESTs were deposited into GenBank without modification. Some ESTs were modified by the submitter to match the genome reference sequences, and some ESTs were actually computationally predicted cDNA from the reference genome. Therefore, we removed the ESTs that did not have any mSNP in order to accurately estimate the contribution of TSV events by allelic gene structure variations. Using the SNP association as illustrated in the upper panel of Fig. 4, more than 28% of transcript variation events were found to be allelic gene structure variations (Fig. 4 lower panel). Interestingly, the proportion of allelic variations is different among different types of TSV. More than half of variations in AltS were from different alleles, while >90% of exon skipping type variations were from alternative splicing.

## Discussion

Good genome annotation is essential to the application of genome sequence data. The utility of *A. gambiae* genome annotation is limited because it is incomplete [8], [10], [20]. In this paper we expanded the *A. gambiae* genome annotation by identifying complete CDS and EST-supported novel genes. We used the new genome annotation to guide the analysis of the transcript structure variations.

Allelic variations are responsible for trait variations in eukaryotic organisms [25]. Gene alleles that generate transcript structure variations are especially interesting since the variations at CDS regions cause more significant changes in protein sequence, structure and function than SNP alone. However, finding such allelic gene structure variations using an empirical approach is difficult because both the genomic sequence and mRNA sequences from one individual are required [6]. A survey of several TSV events indicated that more than 6% of human TSVs were allele-specific based on libraries from human individuals [6], [7]. Unlike human EST and SNP libraries that were generated from a single individual, *A. gambiae* SNP and EST libraries were generated from pooled mRNA of many individuals [8]. Therefore, it is important to develop a general computational approach for *in silico* detection of gene allelic structure variation based on EST sequences alone. The success of our algorithm is based on three assumptions: 1) AS variations come from the same mRNA precursors [26], [27], [28]; therefore transcript structure isoforms from real alternative splicing should have the same SNP patterns; 2) TSVs and SNPs are on the same genes, thus they are linked to each other. The recombination rate in *A. gambiae* was reported to be low, about 1 centimorgan per megabase [29]; and 3) the clones for EST sequencing were selected randomly. As shown in Material and Methods, most EST sequences were obtained from several *A. gambiae* colonies, and each colony was generated from many wild mosquito founders [8], [30], [31]. These data sources benefit our analysis in two ways: 1) since large numbers of ESTs were from limited founders in each colony, real alternative splicing isoforms could be identified; and 2) since the EST data set as a whole was from many colonies that were established from different places, the genetic diversity (alleles) in ESTs is a good representation of the wild *A. gambiae* population.

The distribution of TSV types in *A. gambiae* is very similar to that in *Drosophila*. In *Drosophila*, the most abundant and least abundant TSV types are IntronR and ExonS, respectively. About 31% and 13% of TSV events in *Drosophila* were IntronR and ExonS, respectively [11]. Plants have more IntronR events than insects [16]. Mammals have opposite variation distribution patterns. The most abundant variation types in mammals are ExonS. About 38% to 50% of TSV types are ExonS in humans, and only a small portion of TSVs (∼15%) is IntronR in humans and mice [4], [11]. The TSV distribution is apparently related to the evolutionary phylogenetic tree. We also found that gene allelic variations contribute to transcript structure variations differently in different variation types in *A. gambiae* (Fig. 4). It is interesting that the majority of AltS variations were contributed by allelic variations. We hypothesize that this observation is related to the structure of precursor mRNA. It has been reported that mRNA splicing is affected by the structure of mRNA precursors [8], [32], [33]. The structure changes required for AltS cases are much larger than those for other TSV cases, and these changes are mostly caused by significant precursor sequence alteration. For ExonS, the most abundant TSV type in vertebrates and least abundant TSV type in insects and plants, only a small percentage (<10%) was caused by allelic gene structure variations. On the other hand, for IntronR, the most abundant type in invertebrates and plants and the least abundant type in vertebrates, >30% were from allelic gene structure variations. This result is consistent with the observation that ∼28% of TSVs are from allelic gene structure variations in *A. gambiae* while only 6% of TSVs are from allelic gene structure variations in humans [7]. It suggests that allelic variations contribute to transcript variation more in invertebrates and plants than in vertebrates, while alternative splicing plays a major role in vertebrates.

The allelic gene structure variation mechanism is unknown. Here, we propose two potential mechanisms. First, the deletions/insertions mutations within exon regions simulate IntronR alternative splicing [34]; Second, tandem repeats at processing sites cause allelic structure variations, since tandem repeats are highly unstable [35]. We observed several variations that could be explained with these mechanisms in our data.

In summary, we expanded the genome annotation of *A. gambiae* by increasing the correct CDS boundaries and finding novel genes. About 10% of genes in *A. gambiae* have transcript isoforms. Most of the isoforms were created by intron retention mechanisms. The majority of variations were located in CDS regions, and their open reading frames were conserved between variations. About 28% of transcript structure variations were predicted from different gene alleles. Since mutations in intron regions were invisible in ESTs and some rare SNPs were not shown in mSNP, the number of TSVs contributed by allelic gene variations is expected to be higher than the prediction. Because gene structure variations change the gene products much more significantly than SNPs, the allelic gene structure variations are excellent candidates for the direct and indirect association studies.

## Materials and Methods

### Gene prediction

An AnoGold dataset that contained EST-supported genes was generated [36]. After removal of the incomplete cDNA (missing the start/stop codons) and cDNA not perfectly matched to the reference genome from the AnoGold set, we obtained ∼900 full-length cDNA. We aligned these cDNA to the *A. gambiae* reference genome to extract the intron/exon information, and generated two specific files: a sequence file and a coordinate file. The median size of 5′-UTR and 3′-UTR for this set was 234bp and 451bp respectively. The two files and UTR length values were used to train GlimmerHMM [22], an *ab initio* gene prediction tool for eukaryotes, to get an *A. gambiae* specific gene model. Then we synthesized the gene set from GlimmerHMM and the gene set from vectorbase.org (Oct 2007 release, AgamP3.4) with the combinational prediction method as described previously [20].

### EST data set and reference genome

About 153,165 *Anopheles gambiae* ESTs were downloaded from dbEST, and 62,891 from the CoreNucleotide database. From a major EST project [8], 67,044 ESTs were extracted from GenBank at NCBI based on accession numbers. In addition, we used 608 ESTs from the previous work [37]. Together, 283,708 ESTs were obtained. After removal of the ESTs that have the same accession number, we retained 236,004 *A. gambiae* ESTs as a final set for this project. About 35% of these ESTs were created from the RSP-ST *An. gambiae* mosquito colony [31], ∼28% were from the 6–9 colony [8], ∼12% were from the 4arr colony [30], 2% were from the G3 colony, and the remaining ESTs came from other colonies (such as L35), wild mosquitoes or unknown sources. Each colony was generated from many wild mosquito founders. *Anopheles gambiae* reference genome sequences (AgamP3, February 2006) were downloaded from vectorbase (www.vectorbase.org).

### Transcript structure variation detection

ESTs were aligned to the *A. gambiae* reference genome separately by two sequence alignment tools: GMAP [38] and GeneSeqer [39]. After alignment by each tool, the information on exon, intron, EST gi number, coverage and identity for each EST was extracted and stored in a mysql database. Uniexonic coding sequences were removed to avoid genomic DNA or premature mRNA contamination [40], [41]. The ESTs that were aligned to the genome with high coverage (>80%) and identity (>95%) were used to detect the transcript structure variations using ASpipe [16]. The transcript structure variations were classified into six different categories (Fig 1, upper panel): intron retention (IntronR), alternative acceptor site (AltA), alternative donor site (AltD), alternative acceptor and donor sites (AltS), exon skipping (ExonS), and Others. To remove false positives resulting from the sequence alignment errors, the TSVs that were detected by both GMAP and GeneSeqer were used for further analysis.

### SNP detection

We extracted the SNPs based on alignments between ESTs and the *A. gambiae* reference genome. An EST sequence might match several loci on the genome; however, only the best alignments were used to extract the SNP. The SNPor, software for SNP detection, was developed to parse the alignments to discover the mismatched base pairs between ESTs and the reference genome. For each SNP, its genomic position, nucleotide name, and EST gi number were obtained. Because false positive SNPs could be introduced by sequence alignment errors and sequencing errors, only the SNPs that were present in more than one EST were used to detect allele-specific transcript structure variation in this paper.

### *In silico* detection of allelic gene structure variations from alternative splicing

By definition, the AS isoforms were generated after gene transcription. Therefore, the structure isoforms should share the same SNP pattern. If the SNP pattern is different, the transcripts are from different alleles. Since the transcript structure variation regions and the SNPs are within the same gene, they are tightly linked. The SNPs located at exon regions were thus used as tags to distinguish two transcript structure variation sources: allelic variations and post-transcriptional alternative splicing.

As illustrated in the upper panel of Fig 4, for each variation event, two groups of ESTs were extracted: 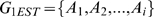, 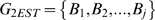. The ESTs in each group had the same transcript structure variation isoform. Each EST in G_1EST_ was compared with every EST in G_2EST_. The overlapping positions of the two ESTs were calculated. The SNP patterns at overlapping regions were obtained to form the following two matrices:
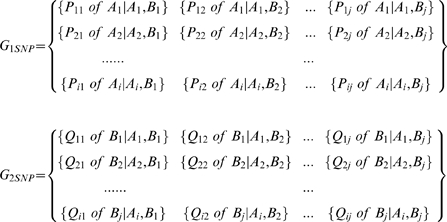
where G_1SNP_ and G_2SNP_ were two groups of SNP patterns corresponding to the two EST groups of G_1EST_ and G_2EST_ respectively. Any matches between P_ij_ from G_1SNP_ and Q_ij_ from G_2SNP_ would classify the variation event source as alternative splicing. If no matches were found between P_ij_ and Q_ij_, the variation source would be gene allelic variation (equation 1).
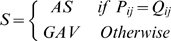
(1)


### Data Availability

All EST data can be obtained from NCBI. The constitutional CDS structures for each gene, the transcript structure variations and gene functions can be downloaded as a spreadsheet from our website at http://www.omics.umn.edu/download/TSV/ or from the NIH website at http://exon.niaid.nih.gov/transcriptome/ReanoXcel-2009/ReanoXcel-2009.zip. The CDS structures can also be viewed at http://www.vectorbase.org by selecting ReAnoCDS from the DAS Sources pull-down menu at the MapView page. The mSNPs reported in this paper have been deposited to the dbSNP database at NCBI (http://www.ncbi.nih.gov). They are available in NCBI (http://www.ncbi.nlm.nih.gov/projects/SNP/snp_viewBatch.cgi?sbid=1050338). The “Submitter handle” for these SNP is OMICSTECH. These mSNPs are also available from the web at http://www.omics.umn.edu/download/TSV/AgSNP10.tab. A web interface for SNPor is available at http://www.omics.umn.edu/research/SNPor.php. And the software is available upon request from the corresponding author.
